# EzMol: A Web Server Wizard for the Rapid Visualization and Image Production of Protein and Nucleic Acid Structures

**DOI:** 10.1016/j.jmb.2018.01.013

**Published:** 2018-07-20

**Authors:** Christopher R. Reynolds, Suhail A. Islam, Michael J.E. Sternberg

**Affiliations:** Centre for Integrative Systems Biology and Bioinformatics, Department of Life Sciences, Imperial College London, Kensington, London SW7 2AZ, UK

**Keywords:** graphics, visualization, EzMol, molecular viewer, Server

## Abstract

EzMol is a molecular visualization Web server in the form of a software wizard, located at http://www.sbg.bio.ic.ac.uk/ezmol/. It is designed for easy and rapid image manipulation and display of protein molecules, and is intended for users who need to quickly produce high-resolution images of protein molecules but do not have the time or inclination to use a software molecular visualization system. EzMol allows the upload of molecular structure files in PDB format to generate a Web page including a representation of the structure that the user can manipulate. EzMol provides intuitive options for chain display, adjusting the color/transparency of residues, side chains and protein surfaces, and for adding labels to residues. The final adjusted protein image can then be downloaded as a high-resolution image. There are a range of applications for rapid protein display, including the illustration of specific areas of a protein structure and the rapid prototyping of images.

## Highlights

• We describe EzMol, a new Web server for the rapid visualization of protein structure.
• A wizard interface leads the user step-by-step through a focused set of options.
• Options are based around the most common requirements for molecular visualization.
• EzMol does not require any software to be downloaded and is cross-browser compatible.
• EzMol is designed for occasional users and does not require commands to be memorized.

## Graphical Abstract

**Figure:**
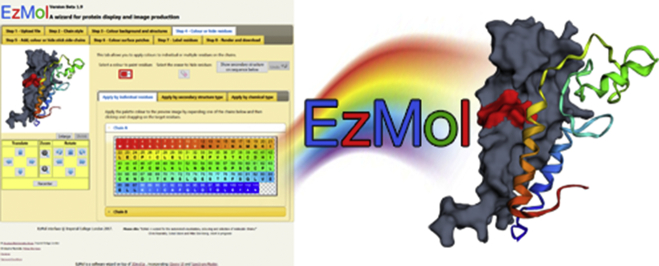

## Introduction

Since the first computerized molecular modeling program in 1966 [1], molecular visualization programs have risen to become ubiquitous within the field of biochemical sciences. Today, dedicated molecular graphics programs such as RasMol [2], PyMol [3] and UCSF Chimera [4] alone have tens of thousands of citations between them. In addition to these, users will typically encounter many viewers associated with servers, for example, the Swiss-PdbViewer [5] on the SWISS-MODEL server [6] or the Molecular Biology Toolkit [7] on the RCSB PDB Web site [8]. Online plugins to display molecules include JMol (and its JavaScript-only version, JSMol) [9] and 3Dmol.js [10]. A review of some other graphics programs is presented in Table S1 of Supplementary Content. However, our experience has convinced us that there is a major requirement for a graphics program that

● is simple to use, with minimal menus and without extensive options that could confuse a user;
● requires no text input (apart from the initial PDB code);
● rapidly performs a carefully identified limited set of tasks addressing the most common molecular visualization requirements;
● acts as a software wizard, leading a user step-by-step through a focused set of options;
● requires no local software other than a Web browser that is HTML5- and JavaScript-enabled;
● is cross-browser compatible on multiple platforms; and
● rapidly produces publication quality images.

These specifications have led to the development of the EzMol Web server, a software wizard designed to guide a user through a series of steps designed to encompass the most common needs for the visualization of protein molecules. EzMol is located at http://www.sbg.bio.ic.ac.uk/ezmol/.

## Why EzMol?

Our work was inspired by RasMol [2] and the impact it has had. In the mid-1990s, Sayle and Milner-White developed RasMol, which opened protein visualization to a wide community by virtue of the lack of requirement for specialist hardware and its easy-to-use interface. According to Google Scholar, the landmark RasMol paper has over 2800 citations.

Since then there have been several graphics programs developed exploiting hardware advances and providing far greater functionality than RasMol (see Table S1 of Supplementary Content). Despite this, a 2013 survey [11] examining a cross-section of graphics users from graduate students to full professors found that 11% of those surveyed still preferred RasMol/RasTop, with JMol (in all forms) being the most popular choice (38%), and then PyMol with 25%. Even in 2017, there remains a relatively wide user-base for RasMol, with 80 citations of its primary reference. This is despite the fact that RasMol is limited to 8-bit graphics and is no longer under development, and with the RasMol host Web page advising users to use other graphics programs [12]. This remaining user-base is possibly because of RasMol's simplicity of use.

Use of the PDB database is increasing year on year, with over 679 million total downloads from the RCSB PDB in 2017 [13], creating a wide need for molecular visualization as this includes a large community of non-experts. We therefore conclude that there is a pressing requirement for a contemporary, Web-based, simple to use graphics program.

## EzMol features and functionality

Table 1 compares features of EzMol with those of JSMol, PyMol and UCSF Chimera. These programs have greater capability, but there is a trade-off between usability and control. To spotlight the practicalities of EzMol, to apply a single application of color to a contiguous set of residues can be done using three clicks in EzMol: two to open the palette and select a color and one to highlight the residues. EzMol also has the benefit of being able to see the color being applied in real time as the corresponding cells are highlighted.

**Table 1:** Program features compared between EzMol, JMol, PyMol and UCSF Chimera

Feature	EzMol	JMol/JSMol	PyMol	UCSF Chimera
User interface	Software wizard	Toolbar and command line	Toolbar and command line	Toolbar and command line
Free for non-commercial use	Yes	Yes	No	Yes
Requires download	No	Yes	Yes	Yes
No installation required	Yes	No (requires Java)	No	No
Open source	No	Yes	No	No
Does not require a manual for menus and syntax	Yes	No	No	No
Click-and-drag real-time coloring	Yes	No	No	No
Ray tracing	No	Model can be exported and then ray traced with POV-Ray [14] or similar.	Native support	Model can be exported and then ray traced.
Animation capabilities	No	Yes	Yes	Yes

EzMol processes files in the Protein Data Bank (PDB) Atomic Coordinate Entry Format [15] and uses them to generate a customized image manipulation tool for that structure.

EzMol is implemented as a Web-based software wizard interface. It is written in HTML and JavaScript and rendered by Perl Scripts as shown in Fig. S1 of Supplementary Content. JQuery plugins are used to display tabs and accordions in the layout.

The key feature of EzMol is that it is a step-by-step wizard-driven program, and accordingly, we will report its features following the steps in order.

The home page of EzMol (see Fig. S2 of Supplementary Content) contains a submission form which gives the user a choice of whether to browse for a PDB file on their local storage to upload (limited to five megabytes in size), browse for a previously stored user-generate EZM file to upload, or enter a PDB ID code and load the coordinates from the EzMol server. EZM files are the format that EzMol allows users to save their work in. They are designed to be human-readable, so that, if necessary, users can edit EZM files in their raw format.

**Fig. 1:**
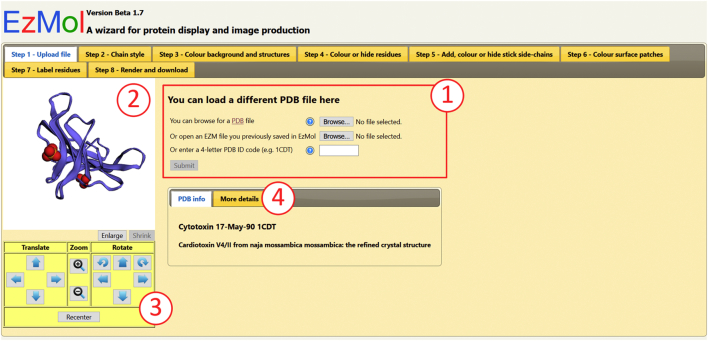
EzMol rendered page, showing Step 1 of the EzMol interface: Upload File.

**Fig. 2:**
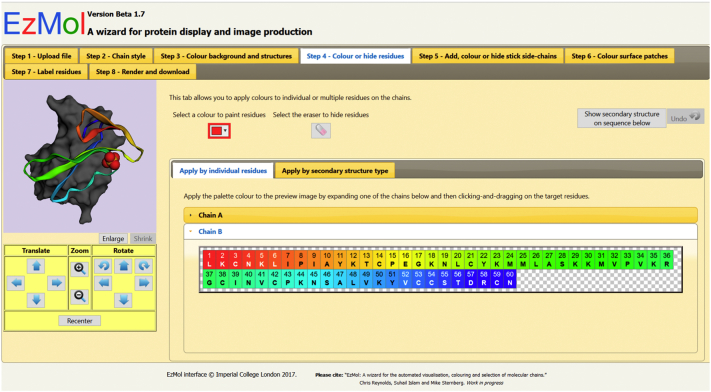
Step 4 of the EzMol interface: color or hide residues.

A rendering script (see Fig. S1 of Supplementary Content) generates the interactive EzMol page from the PDB or EZM file. Figure 1 shows the EzMol display after the user has uploaded a protein coordinate file (in this example, 1CDT [8]). The first tab of the generated page contains a similar submission form to the home page (marked as Number 1 on Fig. 1) to allow the user to upload subsequent files without having to return to the home page. The rendering script generates an interface that displays all the chains and residues (see Fig. 2) and incudes the PDB structural data within the HTML of the generated page. From these data, 3Dmol.js [10] is used to display the PDB data in a structural viewer preview pane (marked as Number 2 on Fig. 1) which can be manipulated with the mouse.

Number 3 on Fig. 1 indicates the movement controls, for translating, zooming and rotating the molecule object. Importantly, the user does not need first to find out and then recall mouse combinations for movement. However, to provide the user with options, these actions can also be performed using the mouse and by holding down the Shift key to zoom and the Ctrl key to translate.

EzMol reads data from the PDB file and displays it in an easy to read format, behind the “More details” tab (Number 4 on Fig. 1).

Figure 2 shows the fourth step, where the user can color chains by individual residues. The display for the residues is controlled by an Amino Acid Grid Selector, and similar ones are used for sticks, surface coloring and labels. The selector is a grid that functions in a similar manner to date selector widgets on Websites that require calendar bookings, allowing the user to click and drag to select residues. Thus, the user does not have to recall command line syntax to select or color residues. The grid represents all amino acids in a chain, with each cell representing an amino acid in the chain. Each cell displays the PDB sequence number, the insertion code (if applicable) and a single-letter code for the amino acid. The background color of the cell indicates the color on the displayed image. Mouse over on a cell also gives the full name of the amino acid. Accordions for each chain can be expanded to show boxes representing all the residues in the chain. In DNA chains, they display the nucleotides. Each box contains the PDB number (and insertion code where relevant) and the one-letter code for the amino acid or nucleotide. The background color of the box represents the coloring displayed on the corresponding areas of the preview. An eraser is provided to erase/hide areas of the image. This is equivalent to setting the areas to a fully transparent color. Colors are applied in real time as they are highlighted in the case of residues and side chains. Surface patch colors are applied only after the mouse-up event.

EzMol also contains an undo function. Figure 2 also shows the undo button in the top-right corner and the “Show secondary structure on sequence below button,” which changes the single-letter amino acid codes on the grid to symbols representing secondary structure that the amino acid is part of (α for alpha helices, β for beta-sheets, · for coils). A second tab allows the user to apply a single color to all residues of a common secondary structure type.

Steps 5, 6 and 7 similarly apply stick side chains, electrostatic surface coloring and labels. They are described in detail in Figs. S7, S8 and S9 of Supplementary Content.

**Fig. 3:**
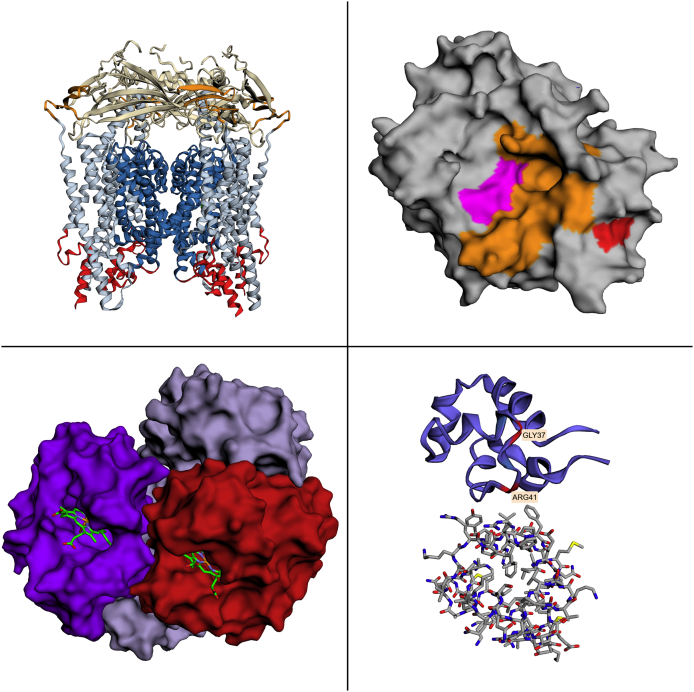
A gallery of example images generated by EzMol. Top left: Ion-channel protein TRPML3, duplicating the coloring shown in Fig. 1b of the *Nature* paper of Hirschi *et al*. [16]. Top right: Transmembrane protease TMPRSS6 modeled using the Phyre [17] structure prediction server duplicating the coloring shown in Fig. 1 of the *Nature Genetics* paper of Chambers *et al*. [18]. Bottom left: PDB structure 1GZX [19], oxy T state hemoglobin; chains displayed as solid structures and haem groups displayed as sticks. Bottom right: PDB structure 3CRO [20], DNA chains hidden, chain L colored the default blue with two residues colored in red and labeled, chain R displayed as a stick structure.

The eighth step, “Render and download,” allows the user to save their work (as a readable file containing all commands applied in the EzMol session) or download the image in a resolution of 1280 pixels square in Portable Network Graphics format. Figure 3 shows four example images that have been generated by EzMol.

## Discussion

To illustrate the power of EzMol and demonstrate that it can be used to generate scientifically useful images that communicate scientifically useful information, we considered two images in papers from high-impact journals. First, the *Nature* paper of Hirschi *et al*. [16] which describes the ion channel protein TRPML3 (PDB accession code 5W3S). To duplicate the coloring shown in Fig. 1b of that paper requires 69 clicks and an EzMol version of this protein is shown in the top left of Fig. 3. Second, we considered the *Nature Genetics* paper of Chambers *et al*. [18] (a paper with 159 citations according to Google Scholar), which describes the human serine 6 transmembrane protease TMPRSS6 (structure not available in the PDB; modeled using the Phyre [17] structure prediction server). To duplicate the coloring shown in Fig. 1 of that paper requires 19 clicks, which highlight catalytic residues (colored magenta in the figure), subsite residues (colored orange) and a missense mutation (colored red) thought to affect hemoglobin level maintenance [18], [21]. An EzMol version of this protein is shown in the top right of Fig. 3.

EzMol is intended to be a simple to use tool to allow the quick rendering and coloring of molecular structures. It provides limited functionality that is nevertheless sufficient for many biological purposes and does not require the user to download software. EzMol has applications in situations where the user has a short time to prepare images for an e-mail, presentation or lecture, or even using it dynamically to highlight areas of structures onscreen and visualize where they lie on the protein structure. For example, this would be helpful in using the program to illustrate the location of residue contacts and the location of residues that have undergone missense mutations. EzMol also has potential for teaching, particularly for younger students who have not yet learned how to use a more complex molecular graphics program, and for the prototyping of macromolecule images.

## Appendix A Supplementary data

Supplementary material Image 1
