# Supplementary material for: EzMol: A Web Server Wizard for the Rapid Visualization and Image Production of Protein and Nucleic Acid Structures
Source: J Mol Biol. 2018 Jul 20;430(15):2244–8. doi: 10.1016/j.jmb.2018.01.013 (PMC5961936; doi:10.1016/j.jmb.2018.01.013)
Supplement: Supplementary file 1 — Supplementary material [file mmc1.docx]

# EzMol Supplementary content

Figure S1 shows the implementation diagram of the EzMol software.


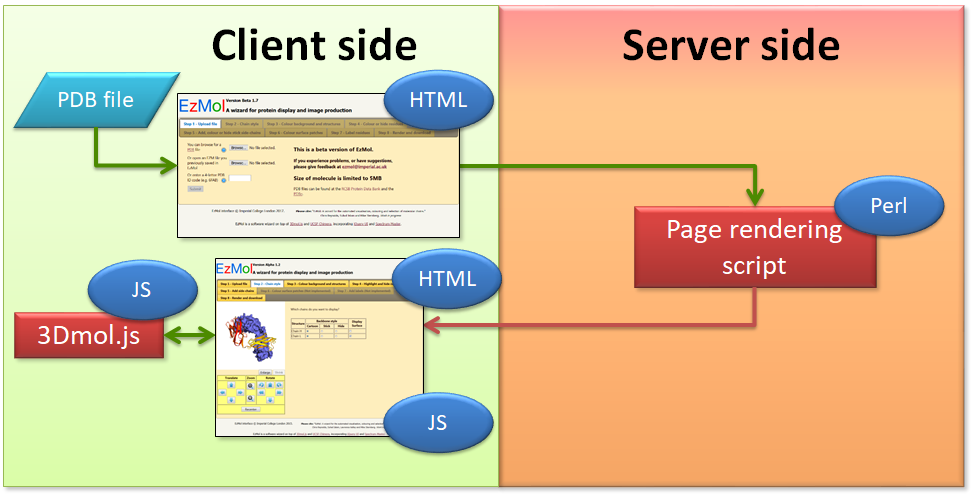


Figure S1. EzMol implementation diagram, showing the interaction of the different stages. The EzMol submission page submits a PDB file, PDB reference number or EZM file to the page rendering script that generates the main EzMol interaction page, that makes use of the 3Dmol.js plugin. Blue ovals show the language that each stage is written in. (JS = JavaScript, HTML = HyperText Markup Language).

# EzMol Wizard Step Guide

1. **Home page/submission page**

Figure S2 shows the home page of EzMol. It contains a submission form which gives the user a choice of whether to browse for a PDB file on their local storage to upload (limited to five megabytes in size), browse for a previously-stored user-generate EZM file to upload, or enter a PDB ID code and load the coordinates from the EzMol server. EZM files are the format that EzMol allows users to save their work in. They are designed to be human-readable, so that, if necessary, users can edit EZM files in their raw format.

Figure S2. The EzMol home page.

1. **Step 1 - Upload file**

The first tab of the generated page contains a similar submission form to the home page (marked as Number 1 on Figure S3) to allow the user to upload subsequent files without having to return to the home page. The rendering script generates an interface that displays all the chains and residues (see Figure S6) and incudes the PDB structural data within the HTML of the generated page. From this data, 3Dmol.js [10] is used to display the PDB data in a structural viewer preview pane (marked as Number 2 on Figure S3) which can be manipulated with the mouse.


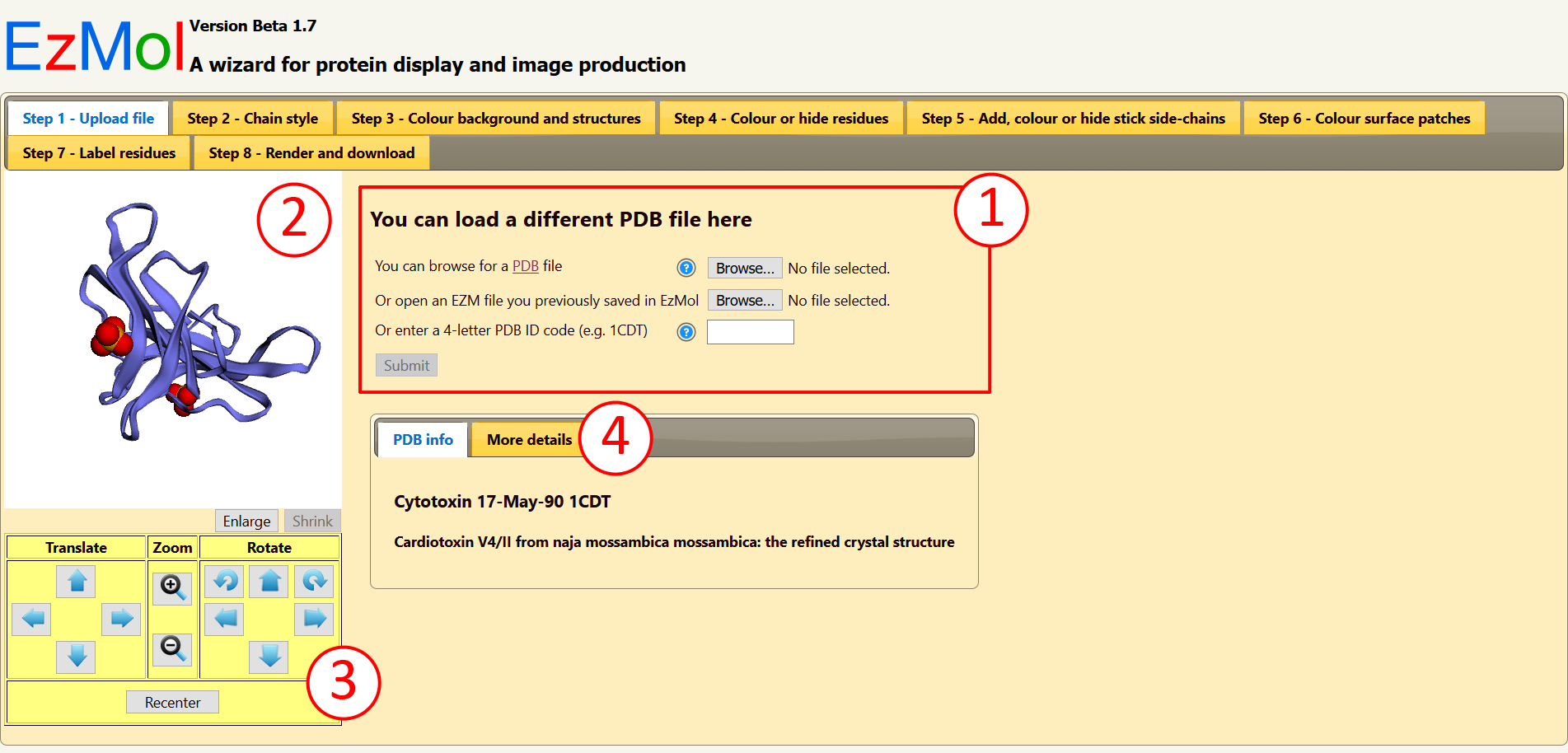


Figure S3. EzMol rendered page, showing Step 1 of the EzMol interface: Upload File.

1. **Step 2 - Chain style**

Figure S4 shows the second step, where the user can select a display style for the chains and the heteroatoms, choosing from cartoon, stick or surface display options. The user can also select to hide structures completely.


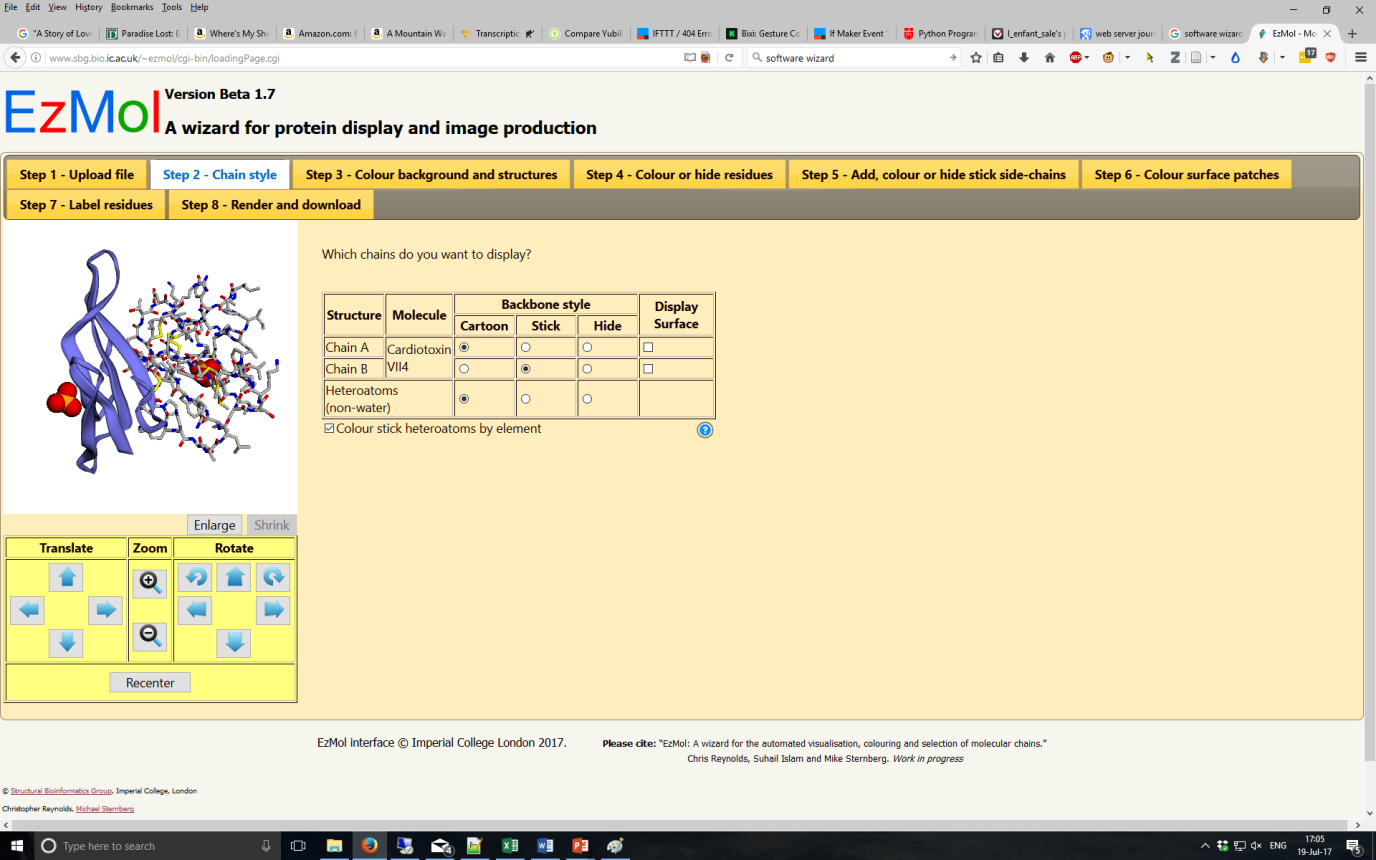


Figure S4. Step 2 of the EzMol interface: Chain Style selection.

1. **Step 3 - Colour background and structures**

Figure S5 shows the third step, “Colour background and structures,” which allows the user to select colours for the chain styles. The Spectrum.js colour picker [1] allows the selection of colours from a palette.


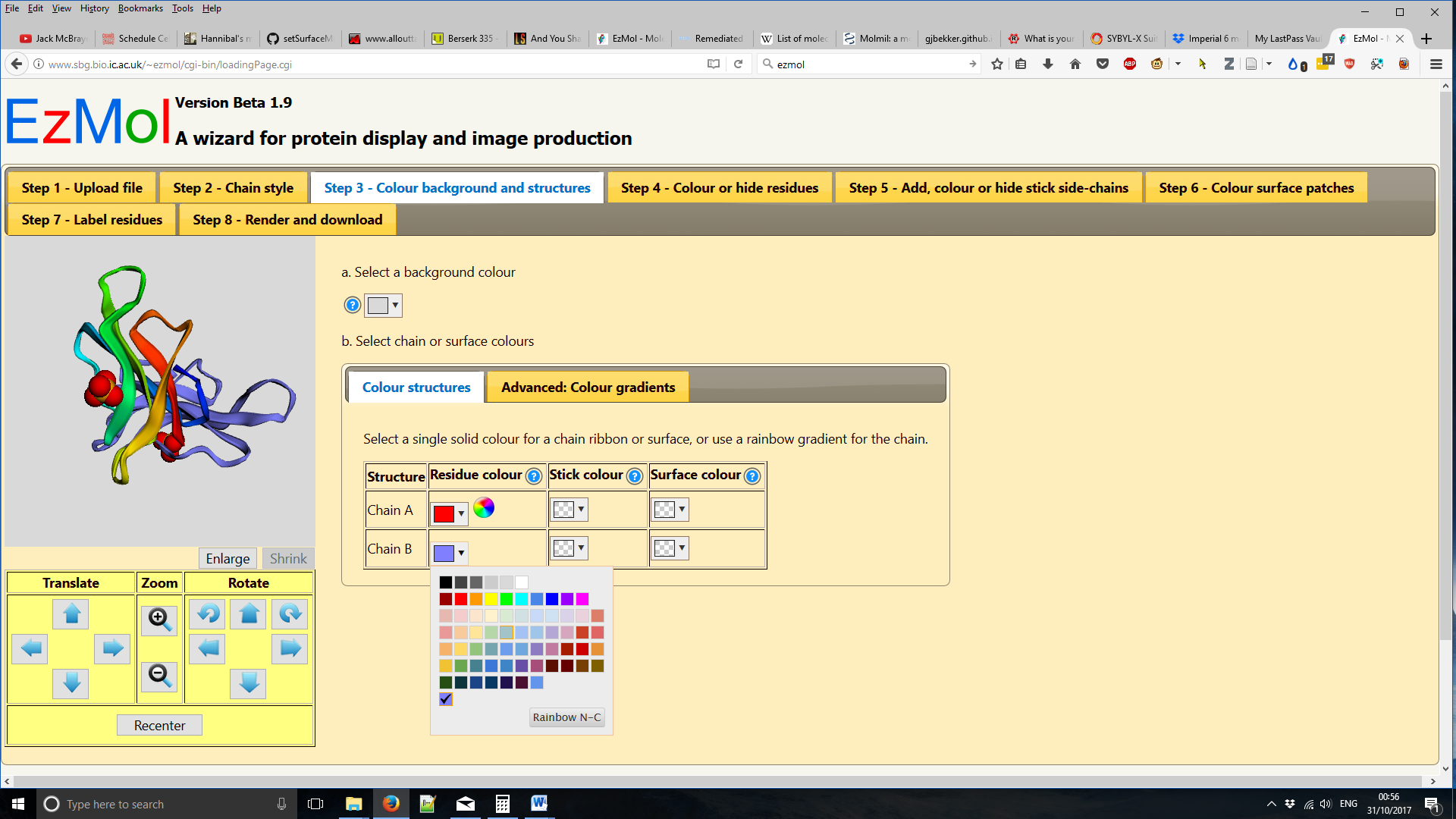


Figure S5. Step 3 of the EzMol interface: Colour background and structures.

1. **Step 4 - Colour or hide cartoons**

Figure S6 shows the fourth step, where the user can colour chains by individual residues. The display for the residues is controlled by an Amino Acid Grid Selector, and similar ones are used for sticks, surface colouring and labels. The selector is a grid that functions in a similar manner to date selector widgets on websites that require calendar bookings, allowing the user to click and drag to select residues. Thus the user does not have to recall command line syntax to select or colour residues. The grid represents all amino acids in a chain, with each cell representing an amino acid in the chain. Each cell displays the PDB sequence number, the insertion code (if applicable), and a single-letter code for the amino acid. The background colour of the cell indicates the colour on the displayed image. Mouse over on a cell also gives the full name of the amino acid. Accordions for each chain can be expanded to show boxes representing all the residues in the chain. In DNA chains, they display the nucleotides. Each box contains the PDB number (and insertion code where relevant) and the one-letter code for the amino acid or nucleotide. The background colour of the box represents the colouring displayed on the corresponding areas of the preview. An eraser is provided to erase/hide areas of the image. This is equivalent to setting the areas to a fully transparent colour. Colours are applied in real time as they are highlighted in the case of residues and side-chains. Surface patch colours are applied only after the mouse-up event.

EzMol also contains an undo function. Figure S6 also shows the undo button in the top-right corner and the “Show secondary structure on sequence below button”, which changes the single-letter amino acid codes on the grid to symbols representing secondary structure that the amino acid is part of (α for alpha helices, β for beta-sheets, **·** for coils). A second tab allows the user to apply a single colour to all residues of a common secondary structure type


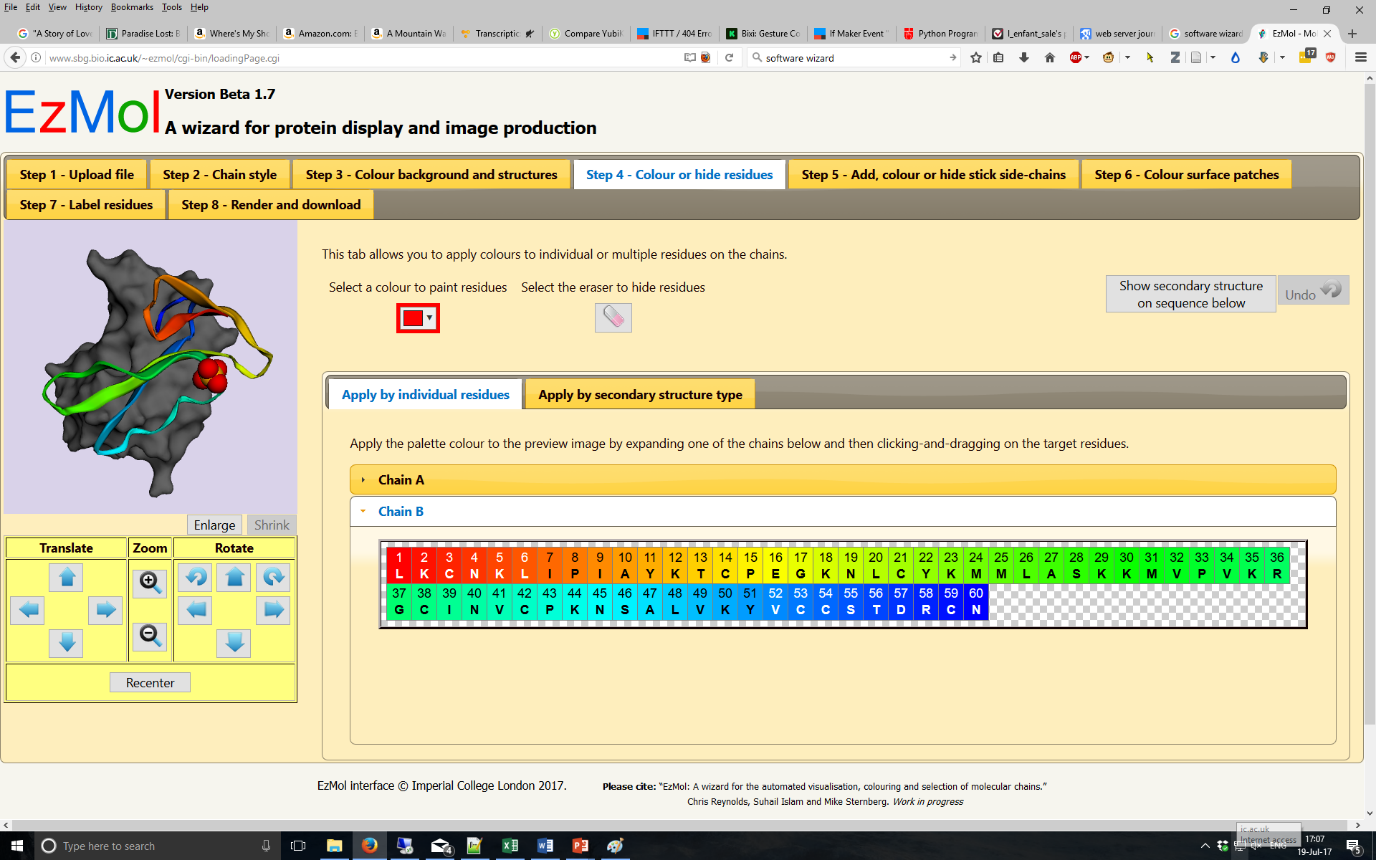


Figure S6. Step 4 of the EzMol interface: Colour or hide residues.

1. **Step 5 - Add, colour or hide stick side-chains**

Figure S7 shows the fifth step, “Add, colour or hide stick side-chains,” which allows the user to select colours for the side chains, and apply side-chains in the selected colour to the chain. Again it uses the Amino Acid Grid Selector interface. Applying side chains to a cartoon ribbon side-chain will display the side-chains attached to the cartoon backbone. Applying side-chains where the cartoon ribbon has been erased will also draw in the stick polypeptide backbone to connect the side-chains rather than display them floating in space. Accordingly, a chain displayed as a stick in Step 2 is equivalent to an entirely erased cartoon chain, and all side-chains coloured. A checkbox allows the user to colour the stick heteroatoms by element (colouring the nitrogen, oxygen, phosphorous and sulphur atoms while keeping the carbons to the user-selected colour). The colouring by heteroatom setting is limited to being uniform for the whole structure.


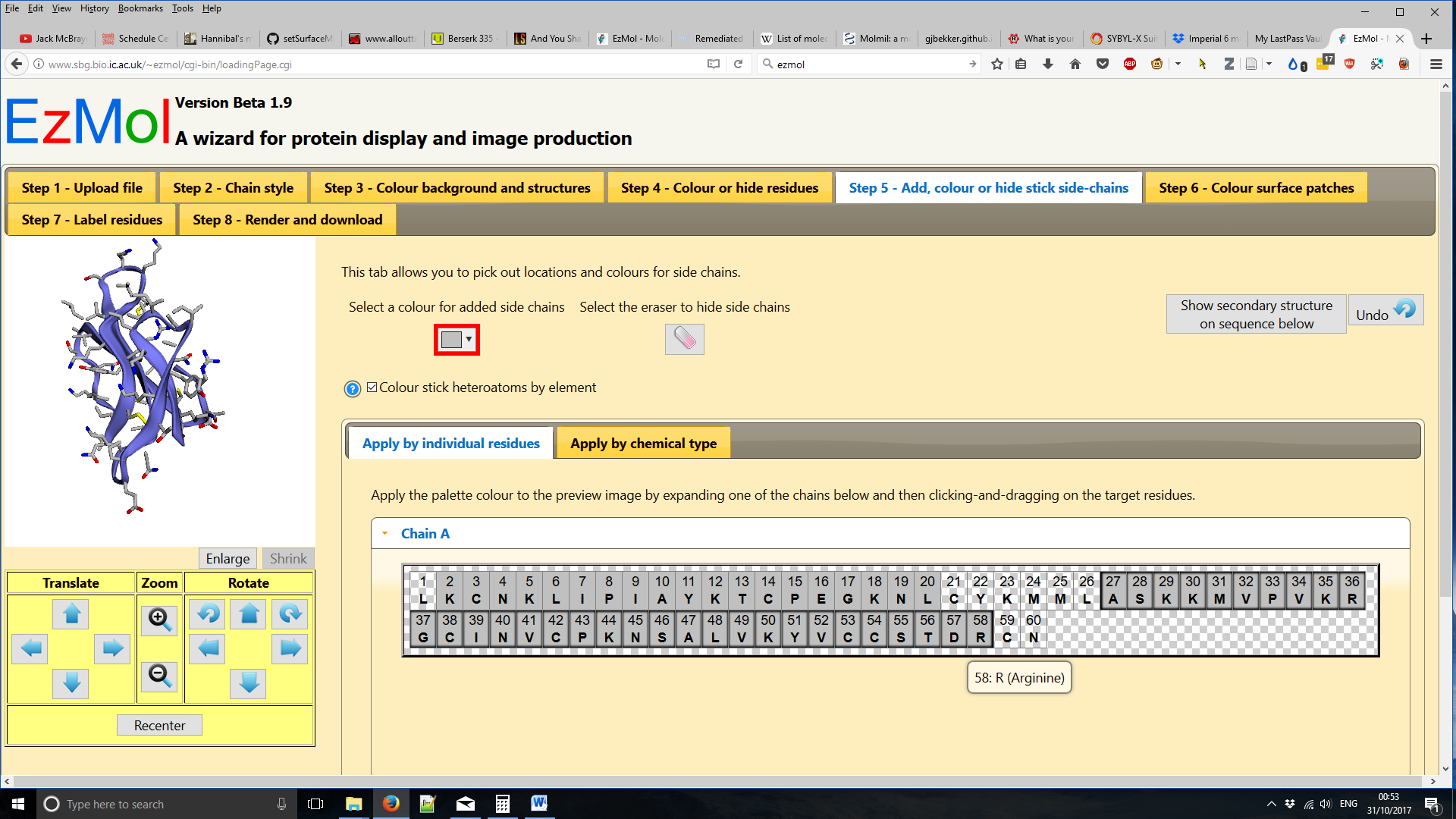


Figure S7. Step 5 of the EzMol interface: Add, colour or hide stick side-chains.

1. **Step 6 - Colour surface patches**

Figure S8 shows the sixth step, “Colour surface patches,” which allows the user to select and apply colours to a solid surface. Again it uses the Amino Acid Grid Selector interface.


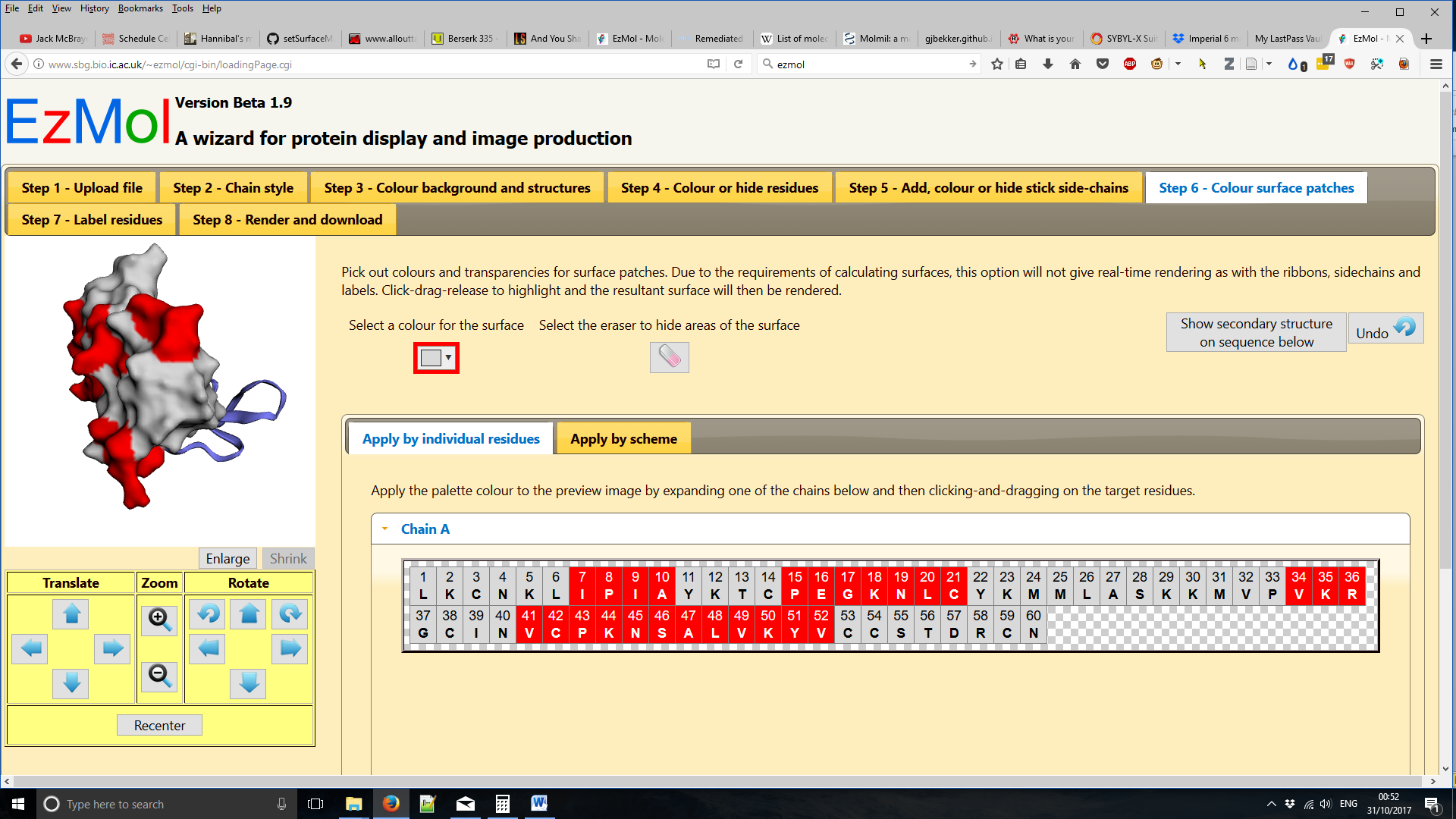


Figure S8. Step 6 of the EzMol interface: Colour surface patches.

1. **Step 7 - Label residues**

Figure S9 of Supplementary Content shows the seventh step, “Label residues”, which allows the user to add labels for the residues onto the image. Using the “Always show labels on top” checkbox, the user has the option of allowing the labels to be hidden by portions of the structure, or to always display them on top of the image. In the “Label style settings” tab, the user can select foreground and background colours for the labels and different ways of displaying the residue name. The label style settings are limited to being uniform for the whole structure.


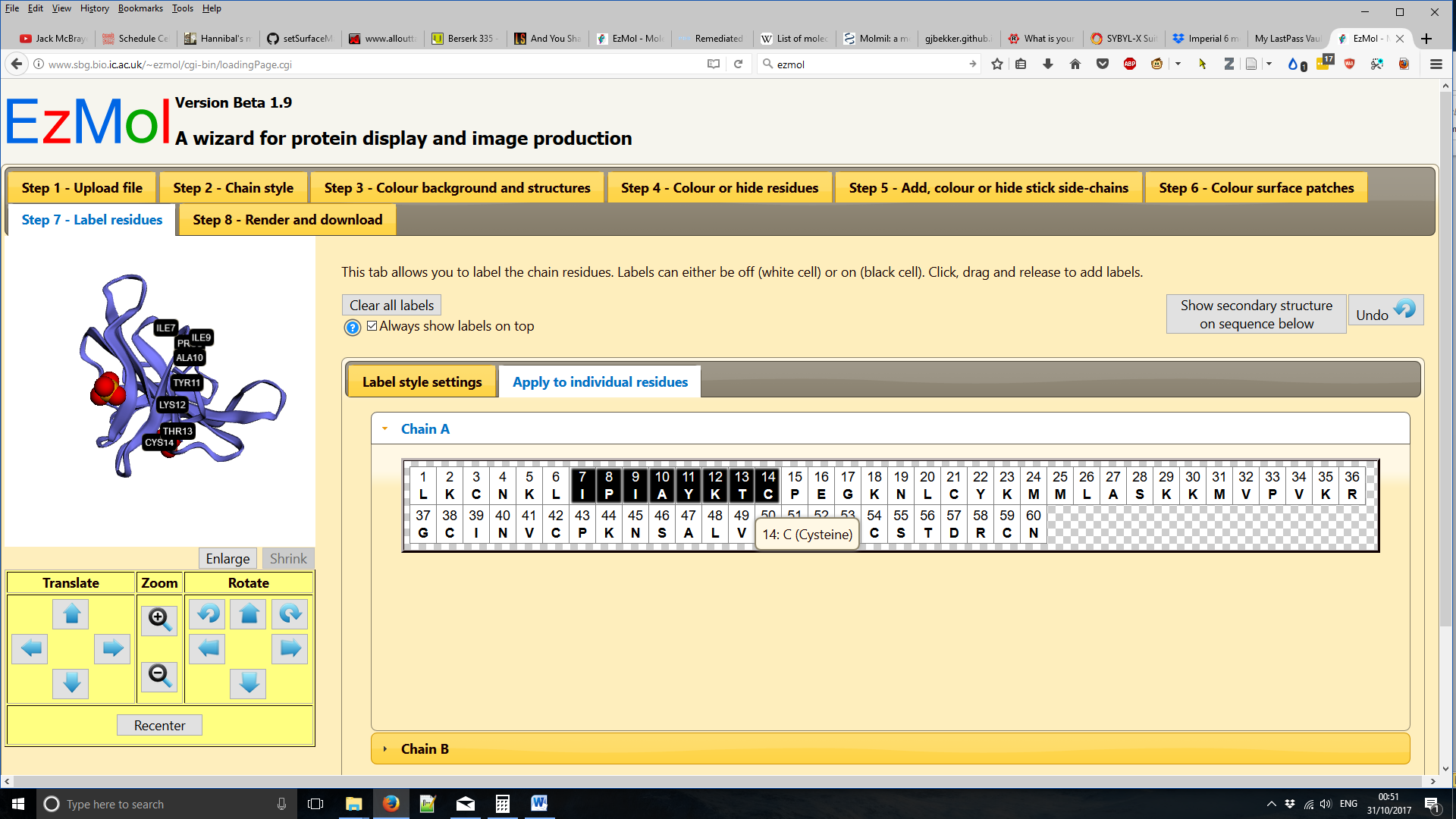


Figure S9. Step 7 of the EzMol interface: Label residues.

1. **Step 8 - Render and download**

The eighth step, “Render and download”, allows the user to save their work (as a readable file containing all commands applied in the EzMol session) or download the image in a resolution of 1280 pixels square in Portable Network Graphics format.

# Other molecular graphics viewers

Table S1 gives a summary of various other available molecular graphics viewers.

Table S1. A table of molecular graphics viewers.

| **Software** | **Description** |
| --- | --- |
| **Web-based software** | |
| 3DBIONOTES [2] | A web server designed to display annotations of biochemical information on protein structures. |
| 3Dmol.js [3] | Free open source molecule viewer web plugin. Used by EzMol. |
| JMol [4] | Free open source molecule viewer web plugin. |
| LiteMol [5] | An online web server for viewing macromoleuar structure. |
| Molmil [6] | Free open source molecule viewer web plugin. |
| POLYVIEW-3D [7] | Online web server that allows users to upload a PDB ID or PDB structure and select from a range of display options to generate a rendered structure. Individual residues can be highlighted through a command-line interface. |
| Swiss-PdbViewer [8] / Deepview | Web plugin used by the SWISS-MODEL server [9]. Quickly generates images of proteins with limited ability for user modification. |
| **Downloadable software** | |
| BioBlender [10] | Open source downloadable software. Based on the dedicated modelling and rendering software Blender. Uses a graphical and command line interface. It has commands to visualise lipophilic and electrostatic potentials. |
| BRAGI [11] | Free downloadable software with a graphical interface. |
| CCP4mg [12] | Free downloadable software that allows the user to select from pre-set templates for molecular visualisations, as well as using a graphical user interface and typed commands to manipulate the structure. |
| Chimera [13] | Dedicated molecular graphics software that can be downloaded and installed. Free for academic or non-profit use. Scriptable. Uses a command-line interface. |
| Cn3D / iCn3D [14] | Free downloadable software, provided by the National Center for Biotechnology, for viewing macromolecular structures on their Entrez database. iCn3D is a web-based plugin version of the software. |
| CrystalMaker [15] | Downloadable software for visualising molecular and crystal structures. Uses a graphical interface. A stripped-down version is free for structures. |
| Kinemage/KING [16] | Free downloadable software for macromolecular visualisation. |
| PyMol [17] | Dedicated molecular graphics software that can be downloaded and installed. Free for educational-use. Scriptable. Uses a command-line interface. |
| MOLMOL [18] | Free downloadable open-source software for analysing and displaying protein and DNA structures, specifically intended for NMR structures. |
| [MolScript](http://www.avatar.se/molscript/) [19] | Free open source software that can be downloaded and installed. Uses a command-line interface. |
| PMV (Python Molecular Viewer) [20] | Free downloadable molecular viewer with a command line interface. There is an embeddable version ePMV (embedded Python Molecular Viewer) for use within 3D animation applications. |
| QuteMol [21] | Open-source downloadable software. User can choose from several pre-set modes to render molecules that can be customised. |
| RasMol [22] | Free downloadable software. Scriptable. Uses a command-line interface. |
| RCSB PDB Viewer / Molecular Biology Toolkit (MBT) [23] | A Java toolkit for building molecular visualisation displays. It is used for visualisations on the RCSB PDB web site [24]. |
| VMD [25] | Downloadable software intended for viewing molecular dynamics simulations. Available in different distributions including one that allows modification of the source code and is free for non-commercial use. |
| YASARA [26] | Downloadable software for visualising proteins. The basic package with visualisation functions is free, with additional purchase required for modules allowing structural and dynamic analysis. |

# References

[1] B. Grinstead, *spectrum: The No Hassle JavaScript Colorpicker*. 2017.

[2] J. Segura *et al.*, “3DBIONOTES v2.0: a web server for the automatic annotation of macromolecular structures,” *Bioinforma. Oxf. Engl.*, vol. 33, no. 22, pp. 3655–3657, Nov. 2017.

[3] N. Rego and D. Koes, “3Dmol.js: molecular visualization with WebGL,” *Bioinforma. Oxf. Engl.*, vol. 31, no. 8, pp. 1322–1324, Apr. 2015.

[4] E. Willighagen and M. Howard, “Fast and Scriptable Molecular Graphics in Web Browsers without Java3D,” *Nat. Preced.*, no. 713, Jun. 2007.

[5] D. Sehnal *et al.*, “LiteMol suite: interactive web-based visualization of large-scale macromolecular structure data,” *Nat. Methods*, vol. 14, no. 12, p. 1121, Dec. 2017.

[6] G.-J. Bekker, H. Nakamura, and A. R. Kinjo, “Molmil: a molecular viewer for the PDB and beyond,” *J. Cheminformatics*, vol. 8, no. 1, Aug. 2016.

[7] A. Porollo and J. Meller, “Versatile annotation and publication quality visualization of protein complexes using POLYVIEW-3D,” *BMC Bioinformatics*, vol. 8, p. 316, Aug. 2007.

[8] N. Guex and M. C. Peitsch, “SWISS-MODEL and the Swiss-Pdb Viewer: An environment for comparative protein modeling,” *Electrophoresis*, vol. 18, no. 15, pp. 2714–2723, Jan. 1997.

[9] T. Schwede, J. Kopp, N. Guex, and M. C. Peitsch, “SWISS-MODEL: an automated protein homology-modeling server,” *Nucleic Acids Res.*, vol. 31, no. 13, pp. 3381–3385, Jul. 2003.

[10] R. M. Andrei *et al.*, “Intuitive representation of surface properties of biomolecules using BioBlender,” *BMC Bioinformatics*, vol. 13, no. 4, p. S16, Mar. 2012.

[11] J. Reichelt, G. Dieterich, M. Kvesic, D. Schomburg, and D. W. Heinz, “BRAGI: linking and visualization of database information in a 3D viewer and modeling tool,” *Bioinforma. Oxf. Engl.*, vol. 21, no. 7, pp. 1291–1293, Apr. 2005.

[12] S. McNicholas, E. Potterton, K. S. Wilson, and M. E. M. Noble, “Presenting your structures: the CCP4mg molecular-graphics software,” *Acta Crystallogr. D Biol. Crystallogr.*, vol. 67, no. 4, pp. 386–394, Apr. 2011.

[13] E. F. Pettersen *et al.*, “UCSF Chimera - A visualization system for exploratory research and analysis,” *J. Comput. Chem.*, vol. 25, no. 13, pp. 1605–1612, Oct. 2004.

[14] Y. Wang *et al.*, “Cn3D: sequence and structure views for Entrez,” *Trends Biochem. Sci.*, vol. 25, no. 6, pp. 300–302, Jun. 2000.

[15] “CrystalMaker Software: Crystal & Molecular Structures Modelling and Diffraction.” [Online]. Available: http://www.crystalmaker.com/. [Accessed: 31-Oct-2017].

[16] V. B. Chen, I. W. Davis, and D. C. Richardson, “KING (Kinemage, Next Generation): A versatile interactive molecular and scientific visualization program,” *Protein Sci. Publ. Protein Soc.*, vol. 18, no. 11, pp. 2403–2409, Nov. 2009.

[17] W. L. DeLano, “The PyMOL molecular graphics system.” 2002.

[18] R. Koradi, M. Billeter, and K. Wüthrich, “MOLMOL: a program for display and analysis of macromolecular structures,” *J. Mol. Graph.*, vol. 14, no. 1, pp. 51–55, 29–32, Feb. 1996.

[19] P. J. Kraulis, “MOLSCRIPT: a program to produce both detailed and schematic plots of protein structures,” *Journal of Applied Crystallography*, 01-Oct-1991. [Online]. Available: http://scripts.iucr.org/cgi-bin/paper?gl0206. [Accessed: 01-Nov-2017].

[20] M. F. Sanner, “Python: a programming language for software integration and development,” *J. Mol. Graph. Model.*, vol. 17, no. 1, pp. 57–61, Feb. 1999.

[21] M. Tarini, P. Cignoni, and C. Montani, “Ambient occlusion and edge cueing to enhance real time molecular visualization,” *IEEE Trans. Vis. Comput. Graph.*, vol. 12, no. 5, pp. 1237–1244, Oct. 2006.

[22] R. A. Sayle and E. J. Milner-White, “RASMOL: biomolecular graphics for all,” *Trends Biochem. Sci.*, vol. 20, no. 9, pp. 374–376, Sep. 1995.

[23] J. L. Moreland, A. Gramada, O. V. Buzko, Q. Zhang, and P. E. Bourne, “The Molecular Biology Toolkit (MBT): a modular platform for developing molecular visualization applications,” *BMC Bioinformatics*, vol. 6, p. 21, Feb. 2005.

[24] H. M. Berman *et al.*, “The Protein Data Bank,” *Acta Crystallogr. D Biol. Crystallogr.*, vol. 58, no. 6, pp. 899–907, May 2002.

[25] W. Humphrey, A. Dalke, and K. Schulten, “VMD: Visual molecular dynamics,” *J. Mol. Graph.*, vol. 14, no. 1, pp. 33–38, Feb. 1996.

[26] E. Krieger and G. Vriend, “YASARA View—molecular graphics for all devices—from smartphones to workstations,” *Bioinformatics*, vol. 30, no. 20, pp. 2981–2982, Oct. 2014.
